# Movement-related beta oscillations show high intra-individual reliability

**DOI:** 10.1016/j.neuroimage.2016.12.025

**Published:** 2017-02-15

**Authors:** Svenja Espenhahn, Archy O. de Berker, Bernadette C.M. van Wijk, Holly E. Rossiter, Nick S. Ward

**Affiliations:** a Sobell Department of Motor Neuroscience and Movement Disorders, Institute of Neurology, University College London, 33 Queen Square, WC1N 3BG London, UK; b Wellcome Trust Centre for Neuroimaging, Institute of Neurology, University College London, 12 Queen Square, WC1N 3BG London, UK; c Department of Neurology, Charité University Medicine, Augustenburger Platz 1, 13353 Berlin, Germany; d Cardiff University Brain Research Imaging Centre, School of Psychology, Cardiff University, Maindy Road, CF24 4HQ Cardiff, UK

**Keywords:** Test-retest reliability, Intraclass correlation coefficient, Beta oscillations, EEG, Sensorimotor cortex

## Abstract

Oscillatory activity in the beta frequency range (15–30 Hz) recorded from human sensorimotor cortex is of increasing interest as a putative biomarker of motor system function and dysfunction. Despite its increasing use in basic and clinical research, surprisingly little is known about the test-retest reliability of spectral power and peak frequency measures of beta oscillatory signals from sensorimotor cortex. Establishing that these beta measures are stable over time in healthy populations is a necessary precursor to their use in the clinic.

Here, we used scalp electroencephalography (EEG) to evaluate intra-individual reliability of beta-band oscillations over six sessions, focusing on changes in beta activity during movement (Movement-Related Beta Desynchronization, MRBD) and after movement termination (Post-Movement Beta Rebound, PMBR). Subjects performed visually-cued unimanual wrist flexion and extension. We assessed Intraclass Correlation Coefficients (ICC) and between-session correlations for spectral power and peak frequency measures of movement-related and resting beta activity. Movement-related and resting beta power from both sensorimotor cortices was highly reliable across sessions. Resting beta power yielded highest reliability (average ICC=0.903), followed by MRBD (average ICC=0.886) and PMBR (average ICC=0.663). Notably, peak frequency measures yielded lower ICC values compared to the assessment of spectral power, particularly for movement-related beta activity (ICC=0.386–0.402). Our data highlight that power measures of movement-related beta oscillations are highly reliable, while corresponding peak frequency measures show greater intra-individual variability across sessions. Importantly, our finding that beta power estimates show high intra-individual reliability over time serves to validate the notion that these measures reflect meaningful individual differences that can be utilised in basic research and clinical studies.

## Highlights

• Movement-related beta-band activity shows high test-retest reliability.
• Spectral power measures are more reliable than the corresponding peak frequencies.
• Peak frequency measures at rest display higher reliability than during movement

## 1 Introduction

Oscillatory activity is ubiquitous in the brain and considered essential for the encoding and processing of information (Buzsáki and Draguhn, 2004). Neuronal oscillations in the beta frequency band (15–30 Hz), prevalent in sensorimotor cortex, are related to motor activity, as supported by a range of electroencephalography (EEG) and magnetoencephalography (MEG) studies showing a modulation of beta oscillations with active and passive movement (Alegre et al., 2002), motor imagery (McFarland et al., 2000, Nakagawa et al., 2011) and movement observation (Babiloni et al., 2002). Beta power decreases just prior to and during movement (Movement-Related Beta Desynchronization, MRBD), followed by a transient post-movement increase above pre-movement levels (Post-Movement Beta Rebound, PMBR) (Pfurtscheller and Lopes Da Silva, 1999, Pfurtscheller et al., 1998a, Salmelin and Hari, 1994, Stancak and Pfurtscheller, 1995), with each of these dynamics differentially modulated by experimental factors (for review see Kilavik et al., 2013; Van Wijk et al., 2012). MRBD is typically observed in both contralateral and ipsilateral sensorimotor cortices during unimanual movements, while PMBR typically shows a contralateral preponderance (Salmelin and Hari, 1994, Stancak and Pfurtscheller, 1995). In addition to changes in power within the beta frequency band, individual peak frequency has been shown to be a behaviourally meaningful parameter of oscillatory activity (Kilavik et al., 2012) that differs across regions within the sensorimotor cortex (Salmelin and Hari, 1994), and which is of increasing interest considering recent attention on extrinsic neurostimulation approaches for modulating motor outputs (Guerra et al., 2016, Joundi et al., 2012, Pogosyan et al., 2009). However, despite extensive research, the functional relevance of beta oscillatory activity is still debated (Engel and Fries, 2010, Jenkinson and Brown, 2011, Pfurtscheller et al., 1996).

Direct manipulation of beta oscillations through the application of transcranial alternating current stimulation (tACS) at beta frequency can produce a slowing of movements (Joundi et al., 2012, Pogosyan et al., 2009) suggesting a causal role of sensorimotor beta oscillatory activity in motor control. Alterations in beta activity are also observed in disease states such as stroke (Rossiter et al., 2014a) and Parkinson's disease (Brown, 2007, Heida et al., 2014, Heinrichs-Graham et al., 2013, Little and Brown, 2014). Both patient populations show a reduction in the amplitude of MRBD together with deficits in some aspects of motor control, suggesting that MRBD may be a general assay of the state of the motor system, irrespective of the underlying pathophysiology. In addition, changes in beta oscillations have been observed with ageing, with resting beta power increasing as a function of age (Rossiter et al., 2014b; Heinrichs-Graham and Wilson, 2016), and the amplitude of MRBD and PMBR increasing during development (Gaetz et al., 2010).

Given its potential role as neurophysiological marker of motor system function and dysfunction, rhythmic activity at beta frequencies has received considerable interest in both basic and clinical research (Nicolo et al., 2015, Takemi et al., 2015, Ward, 2015, Wu et al., 2015). Measurements of beta activity may provide insight into the dynamics of disease, potentially providing a clinically relevant biomarker. However, despite prevalent use of EEG/MEG to explore beta oscillatory dynamics in normal brain functioning and pathology, to the best of our knowledge, no studies have systematically assessed their test-retest reliability across multiple recordings. If measures of beta oscillations in healthy individuals are highly variable between separate sessions (high intra-individual variability), EEG assays of beta oscillatory activity are unlikely to be useful as biomarkers (Mayeux, 2004). Reliable spectral estimates of oscillatory activity are therefore a prerequisite for studies designed to test longitudinal changes in clinical and non-clinical populations or therapeutic interventions.

In the current study, we comprehensively assessed the test-retest reliability of spectral power and peak frequency measures of movement-related beta activity in a group of healthy subjects across several weeks. Since MRBD and PMBR estimates quantify movement-related changes in beta power *relative* to a pre-movement (resting) baseline, and recent work by Heinrichs-Graham and colleagues (Heinrichs-Graham and Wilson, 2016) suggests a direct relationship between MRBD and pre-movement baseline beta activity, we also evaluated the reliability of beta oscillations during the pre-movement (resting) baseline period of our motor task. For measures of beta oscillations to be reliable and therefore useful biomarkers in basic and clinical research it is essential that these measures (I) display small within-subject variability and (II) do not change as a function of between-session time interval.

## 2 Methods

### 2.1 Subjects

Six healthy subjects (3 females, mean age ±SD=27±4.7 years) took part in our study to assess the test-retest reliability of movement-related beta oscillations over six EEG sessions (S1–S6 in Fig. 3). The time interval between sessions varied from one week for the first five sessions (range=5–9 days, mean between-session time interval ±SD=7±1 days) to six weeks between the fifth and sixth EEG session (range=39–50 days, mean between-session time interval ±SD=43±4 days). This interval design was chosen to test for a systematic influence of interval length on test-retest reliability. All subjects were right-handed according to the Edinburgh Handedness Inventory (Oldfield, 1971), had normal or corrected-to-normal vision, and fulfilled the following inclusion criteria: (a) no history of neurological or psychiatric disease; (b) no physical disability of the arms or wrists; and (c) no use of drugs affecting the central nervous system or self-reported abuse of any drugs. The study was approved by the National Hospital for Neurology and Neurosurgery, UCL Hospitals NHS Foundation Trust and the local research ethics committee at University College London where the study was conducted. All subjects gave written informed consent in accordance with the Declaration of Helsinki. To minimize circadian fluctuations in beta oscillatory levels (Toth et al., 2007, Wilson et al., 2014), all subjects were tested in the time between 9 a.m. and 1 p.m.

### 2.2 Experimental setup

**Fig. 1:**
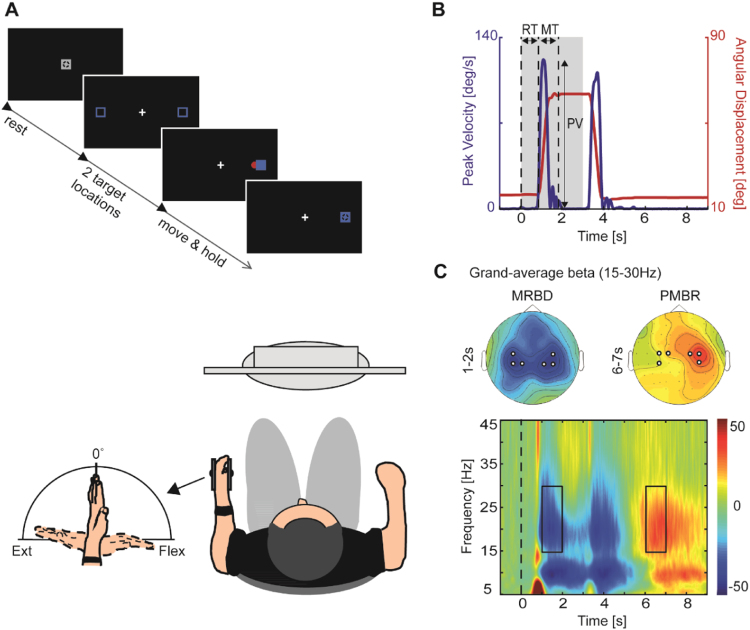
Experimental setup and measurements. A, Experimental paradigm. Subjects sat in front of a computer monitor and were instructed to perform wrist movements to move the wrist cursor (red circle) from the initial start position (grey square) to one of two target positions (blue squares) upon target presentation. B, Calculation of reaction time (RT), movement time (MT) and peak velocity (PV) where the grey patch represents target presentation. Velocity profile (blue line) and wrist angular displacement (red line) are shown for one trial of an example participant. C, Topographical distribution (top panel) and time-frequency map (bottom panel) of movement-related beta activity. Topographical plots of grand-average beta power revealed electrodes of peak change (highlighted as black-and-white disks) overlying contra- and ipsilateral sensorimotor cortices. Time-frequency map for pooled electrodes contralateral to moving hand showing two distinct time windows of peak changes in beta activity (MRBD: 1–2 s; PMBR: 6–7 s). (For interpretation of the references to color in this figure legend, the reader is referred to the web version of this article).

Subjects performed visually-cued wrist flexion and extension with their non-dominant (left) hand rested in an instrumented wrist rig (modified from (Turk et al., 2008)) during EEG recording. The wrist rig consisted of a moulded splint lined with an inflatable pouch and restricted movement to flexion and extension at the wrist joint in the horizontal plane (Fig. 1A, lower panel). The forearm was strapped to a cushioned arm support with the shoulder joint in neutral position and the elbow joint angle between 80° and 90° of flexion. Wrist angular displacement was sensed by a built-in potentiometer, fixed with its axis coaxial to the axis of rotation of the wrist joint. A displacement of 0° indicated a neutral position of the wrist, with the hand being in the same plane as the forearm. The angular position of the wrist was continuously displayed on the computer monitor as a cursor in the form of a red circle – hereafter referred to as “wrist cursor”. On the first day, prior to the task, subjects were instructed on how to perform the motor task and their maximum Active Range Of Motion (AROM) around the wrist joint was measured. The individual maxima and mid-point of AROM were used as target and start position, respectively. During each trial, wrist movements were always initiated from the same start position located at the centre of the screen which represented the subject's individual AROM mid-point. The cue to perform wrist flexion or extension movements was the random appearance of one of two targets (in blue) located equidistant from the central start position (Fig. 1A, upper panel). Each of the targets represented the subject's maximum wrist flexion or extension position. This was designed to ensure the movement distance in each condition was the same; however, the actual movement distance between subjects was different based on their AROM. Subjects were instructed to move the wrist upon presentation of the target so as to shift the red wrist cursor from the central start position to match the position of the target in a quick and discrete movement. They were also asked to move as soon as possible and to avoid anticipation or guessing of target appearance. The target position was displayed for 3 s and subjects had to maintain the wrist cursor inside the blue target until being cued to return to the initial start position. Once subjects returned to the start position, the next cue to move was delivered following a delay of 7±1 s. The task comprised 120 trials (60 trials for flexion and extension, respectively), and subjects were instructed to minimize eye movements by focusing on a centrally located fixation cross.

### 2.3 Data recording

The angular position of the non-dominant wrist was sampled at 100 Hz and sent to the computer for storage and offline analysis. Scalp EEG was continuously recorded at 2084 Hz by 64 electrodes mounted on an elastic cap. Electrodes were evenly distributed over the scalp according to the international 10–20 EEG system (ANT Neuro, Asalab, The Netherlands). The impedance was kept below ≤5 kΩ and the EEG signal was re-referenced to Cz during recording. The timing of the visual cue (blue target) in the motor task was marked in the simultaneous EEG recording, with separate markers for each condition (flexion, extension). Muscle activity was monitored by surface electromyography (EMG) using bipolar electrodes in a belly-tendon montage placed on the wrist extensor (extensor carpi radialis longus) and flexor (flexor carpi radialis) muscles of the non-dominant arm. The raw EMG signal was amplified and band-pass filtered (10 Hz to 500 Hz; D360 amplifier, Digitimer, Hertfordshire, UK) and digitized at an A/D rate of 1 kHz per channel (CED Micro 1401, Cambridge Electronic Design, Cambridgeshire, UK).

### 2.4 Behavioural analysis

Kinematic data were analysed using custom-written routines in Matlab (The MathWorks, Natick, MA). The angular position of the wrist was filtered with a second-order zero-phase shift, low-pass Butterworth filter (cut-off frequency of 10 Hz) and differentiated to calculate velocity. Movement onset was defined as the time when the velocity of the wrist exceeded a threshold of 5% of the maximum velocity and sustained this speed for at least 100 ms. Movement termination was defined as the time when the velocity fell below the threshold for that trial for at least 500 ms. For each subject, trials in which the movement was initiated before the cue signal, reaction time was excessively long (>mean±2.5SD), or movement time was excessively long (>mean±2.5SD) were discarded (average ~7% of trials). Reaction time (RT, interval between visual cue and movement onset), movement time (MT, interval between movement onset and movement termination), and peak velocity (PV) were calculated on the remaining trials (average 111±2) for each individual trial (Fig. 1B) and then averaged within each subject for each experimental condition.

### 2.5 EEG pre-processing and time-frequency transformation

EEG data pre-processing and time-frequency analysis were performed using SPM12 (Wellcome Trust Centre for Neuroimaging, http://fil.ion.ucl.ac.uk/spm) and additional scripts written in Matlab. The raw EEG signal was first offline re-referenced to the average signal across all electrodes, bandpass filtered between 5 and 100 Hz, additionally filtered with a 50 Hz notch filter to reduce line noise contamination, and downsampled to 300 Hz. Data were epoched from -1 to 9 s relative to visual cue onset (0 s). Poorly performed trials (see 2.4 Behavioural analysis) were excluded and the remaining EEG trials were visually scrutinized. Trials containing artefacts (e.g. muscle activation or large eye blinks) were additionally removed. For each session, on average 92±10 artefact-free EEG trials remained for further analyses, and number of trials did not differ between conditions (*p*>0.4) or sessions (*p*>0.1, repeated-measures ANOVA). Artefact-free EEG time-series from each single trial were decomposed into their time-frequency representations in the 5–45 Hz range with frequency steps of 0.1 Hz. A 7-cycle Morlet wavelet was used for the continuous wavelet transformation. Power was averaged across trials and rescaled in order to show changes in power (*P*) relative to the corresponding pre-movement baseline period (-1–0 s prior to cue onset), expressed as percentages of this baseline power (*P*_*ref*_):

$$\%\mathrm{power}=\frac{P-P_{\mathrm{ref}}}{P_{\mathrm{ref}}}*100$$

### 2.6 Spectral power and peak frequency

To select electrodes and time-frequency windows of interest that were orthogonal to potential differences between sessions and conditions, we first examined activity in the a priori chosen beta frequency band (15–30 Hz, (Jurkiewicz et al., 2006; Van Wijk et al., 2012; Yamawaki et al., 2008)), grand-averaged over subjects, sessions and conditions. We then selected electrodes of peak change in beta oscillations from topographical distributions of normalized power (% power), plotted for several time points after cue onset. The topographical maps revealed clear movement-related beta activity (Movement-Related Beta Desynchronization – MRBD; Post-Movement Beta Rebound - PMBR) overlying the sensorimotor cortices, both contralateral and ipsilateral to the moving hand (Fig. 1C; MRBD: ‘C4’ ‘CP4’ ‘CP2’ and ‘C3’ ‘CP3’ ‘CP1’ for contra- and ipsilateral hemispheres, respectively; PMBR: ‘C2’ ‘C4’ ‘CP4’ and ‘C1’ ‘C3’ ‘CP3’ for contra- and ipsilateral hemispheres, respectively). These bilateral electrodes were pooled as contra- and ipsilateral regions of interest, respectively. Note that PMBR was located slightly more anterior to the central midline than the MRBD, consistent with previous EEG (Pfurtscheller et al., 1996) and MEG (Salmelin and Hari, 1994) studies. Next, time-frequency windows were chosen based on peak changes in beta activity in time-frequency maps of these bilateral sensorimotor regions, which revealed clear movement-related beta-band (15–30 Hz) activity in two distinct time windows of interest (Fig. 1C). This information was used to optimize the alignment of constant duration (1 s) time windows to capture maximum MRBD (1–2 s relative to cue onset), occurring between cue onset and movement termination, and PMBR (6–7 s relative to cue onset), which emerges after movement termination. Selected time-frequency windows and electrodes applied to all subjects and sessions, and were not adjusted individually.

Subsequently, for each individual subject, session and condition, mean percentage decrease (MRBD) and increase (PMBR) in beta power were extracted from the respective 1 s time windows and averaged over the pre-selected electrodes for each hemisphere. The absolute pre-movement (resting) baseline beta (BB) power from -1 to 0 s relative to cue onset was also obtained and assessed for reliability.

In addition, individual beta peak frequency was determined semi-automatically for each corresponding time window (BB: -1–0 s; MRBD: 1–2 s, PMBR: 6–7 s). The peak frequency for the MRBD and PMBR were determined as the frequencies having the largest change in spectral power compared to baseline beta power. For the absolute power of baseline beta (BB), first the 1/f shape of the power spectrum was eliminated by fitting and subsequent subtraction of a straight line after log-log transformation (see e.g. Nikulin and Brismar, 2006, Fig. 2). All peaks were selected from the 15–30 Hz frequency range with 0.1 Hz resolution. Cases where no clear peak was present (e.g. Subject 5 Session 1 contra- and ipsilateral hemisphere, and Session 2 contralateral hemisphere), were left out of the analyses.

In total, 12 different beta parameter estimates were used for subsequent analysis: pre-movement beta baseline (absolute power and peak frequency), MRBD (relative power and peak frequency) and PMBR (relative power and peak frequency) from contra- and ipsilateral sensorimotor cortices, respectively.

### 2.7 Statistical analysis

Statistical analyses were performed using SPSS and custom-written Matlab routines. Separate repeated-measures ANOVAs were used to test for differences between sessions, hemispheres and conditions for each of the beta parameter estimates, with ‘time’ (6 levels: sessions 1–6), ‘hemisphere’ (2 levels: contralateral, ipsilateral), and ‘condition’ (2 levels: flexion, extension) as within-subject factors. A Greenhouse-Geiger correction was applied whenever Mauchly's test indicated a lack of sphericity. Post hoc Bonferroni-adjusted paired-samples *t*-tests were performed whenever a main effect was detected. Prior to ANOVA and paired-samples *t*-tests, Kolmogorov-Smirnov test was used to assess normality. All beta parameter estimates and kinematic measures were normally distributed.

The main focus of the statistical analysis was to determine the reproducibility of absolute and relative beta power parameter estimates as well as their corresponding peak frequencies. For this, Pearson correlations were used to assess reliability between two EEG sessions, while Intraclass Correlation Coefficients (ICC) (McGraw and Wong, 1996, Shrout and Fleiss, 1979), based on two-way random effects analysis of variance, were computed to assess the degree of consistency between all six sessions. The ICC assesses the reliability of repeated measures of an individual's beta parameters by comparing the proportion of within-subject variability to all sources of variance; thus, a high ICC value means that within-subject variability is low and that most of the variance is caused by differences between subjects. Following Landis and Koch (1977) suggestions, ICC was rated on the following agreement level: 0.2–0.4 fair, 0.4–0.6 moderate, 0.6–0.8 substantial and >0.8 almost perfect. ICCs were assessed for both movement-related and absolute pre-movement baseline beta activity derived from both sensorimotor cortices. To account for multiple comparisons in the ICC analysis, the significance level was Bonferroni-corrected (corrected *p* values: 0.05/12 for beta parameter estimates and 0.05/6 for kinematic measures).

## 3 Results

### 3.1 Behavioural results

All subjects were able to perform the motor task. The kinematic measures are summarised in Table 1 for each of the six EEG sessions. As expected, reaction time (RT), movement time (MT) and peak velocity (PV) in the motor task were stable across separate sessions, as confirmed by a lack of main effect of ‘time’ for all kinematic measures [RT: *F*_(5,20)_=2.242, *p*=0.156; MT: *F*_(5,20)_=3.661, *p*=0.087; PV: *F*_(5,20)_=0.414, *p*=0.709, all Greenhouse-Geisser corrected]. Subjects performed flexion and extension with similar kinematics [RT: *F*_(1,4)_=0.714, *p*=0.446; MT: *F*_(1,4)_=5.243, *p*=0.084; PV: *F*_(1,4)_=0.771, *p*=0.430] and no significant interactions between ‘time’ and ‘condition’ were found [RT: F_(5,20)_=1.29, *p*=0.328; MT: F_(5,20)_=2.37, *p*=0.159; PV: F_(5,20)_=3.12, *p*=0.090, all Greenhouse-Geisser corrected]. Since there was no significant difference between conditions (flexion, extension), the subsequent results are based on kinematic data collapsed across conditions. Reliability analysis across sessions revealed ICCs of fair to substantial agreement [ICC_RT_=0.750, *p*<0.0001, ICC_MT_=0.370, *p*=0.002], with peak velocity demonstrating highest intra-individual reliability [ICC_PV_=0.774, *p*<0.0001]. This suggests that movement execution remained similar across sessions and that significant neurophysiological differences between sessions cannot be explained by changes in movement kinematics.

### 3.2 Spectral power and peak frequency

**Fig. 3:**
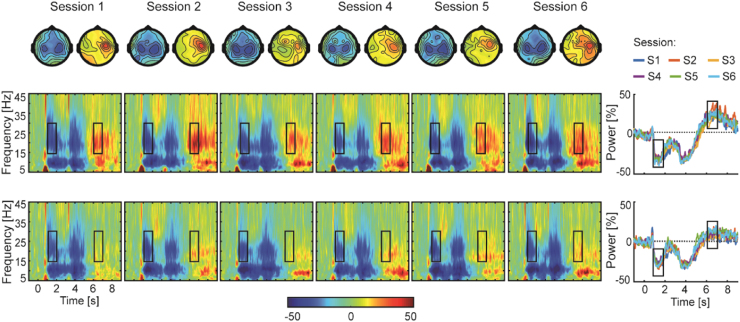
Average movement-related changes in spectral power for each EEG session. Topographies of relative power change in beta frequency (15–30 Hz) during and after movement are averaged over the time window of interest 1–2 s and 6–7 s for MRBD and PMBR, respectively, as indicated by the black rectangles. Time-frequency spectrograms are averaged across subjects separately for contralateral (upper panel) and ipsilateral (lower panel) sensorimotor cortex for all EEG sessions. The right hand panel displays overlaid beta power traces for the six sessions (S1=light blue, S2=orange, S3=yellow, S4=purple, S5=green, S6=blue). The black rectangles indicate the time window of interest (MRBD and PMBR) that were tested for significant differences between sessions and hemispheres. (For interpretation of the references to color in this figure legend, the reader is referred to the web version of this article).

Average spectral changes in contralateral and ipsilateral sensorimotor cortices in response to cue presentation are shown in Fig. 3 for each EEG session. After cue onset and during movement, a reduction in beta power, MRBD, was observed in both sensorimotor cortices with two distinguishable troughs: the first during the movement towards the target and the second during the return to the initial start position. During the static contraction/holding phase of the motor task the strength of beta power increased. This is in agreement with studies demonstrating an increase in beta power as soon as the contraction becomes stable (Baker et al., 1999) or the movement is sustained (Cassim et al., 2000) in line with the hypothesis that beta oscillations play a role in stabilizing the current motor state whilst compromising initiation of new movements (Engel and Fries, 2010, Gilbertson et al., 2005, Van Wijk et al., 2009). After return movement cessation, a strong but transient increase in beta power, PMBR, was observed predominantly in contralateral sensorimotor cortex. The gross morphology of the pattern of movement-related beta oscillations in both sensorimotor cortices shows good resemblance between shorter and longer between-session time intervals.

Estimates of power change during movement (MRBD) and after movement cessation (PMBR) were both unaffected by ‘time’ or ‘condition’ (F-statistics and p-values of all ANOVAs are summarized in Table 2). In addition, while no main effect of ‘hemisphere’ on the magnitude of MRBD was found, PMBR was significantly stronger in contralateral than ipsilateral sensorimotor cortex [F_(1,5)_=7.03, p=0.045, effect size ƞ_p_^2^=0.584], indicating contralateral predominance of the beta power rebound. Throughout the pre-movement baseline period, absolute power estimates were similar across all sessions, conditions and both sensorimotor cortices. Likewise, no significant ‘time x condition’, ‘time×hemisphere’, ‘hemisphere×condition’ or ‘time×hemisphere×condition’ interaction effects were found for any of the spectral power measures (refer to Table 2).

Peak frequency of beta activity in the pre-movement baseline period as well as in the time window in which MRBD occurred did not differ significantly within subjects between sessions, conditions or hemispheres. In contrast, PMBR peak frequency varied as a function of ‘time’ (F_(5,25)_=2.70, p=0.044, effect size ƞ_p_^2^=0.351), but not ‘condition’ or ‘hemisphere’. Finally, there were no significant interactions for any of the peak frequency measures (Table 2).

**Fig. 4:**
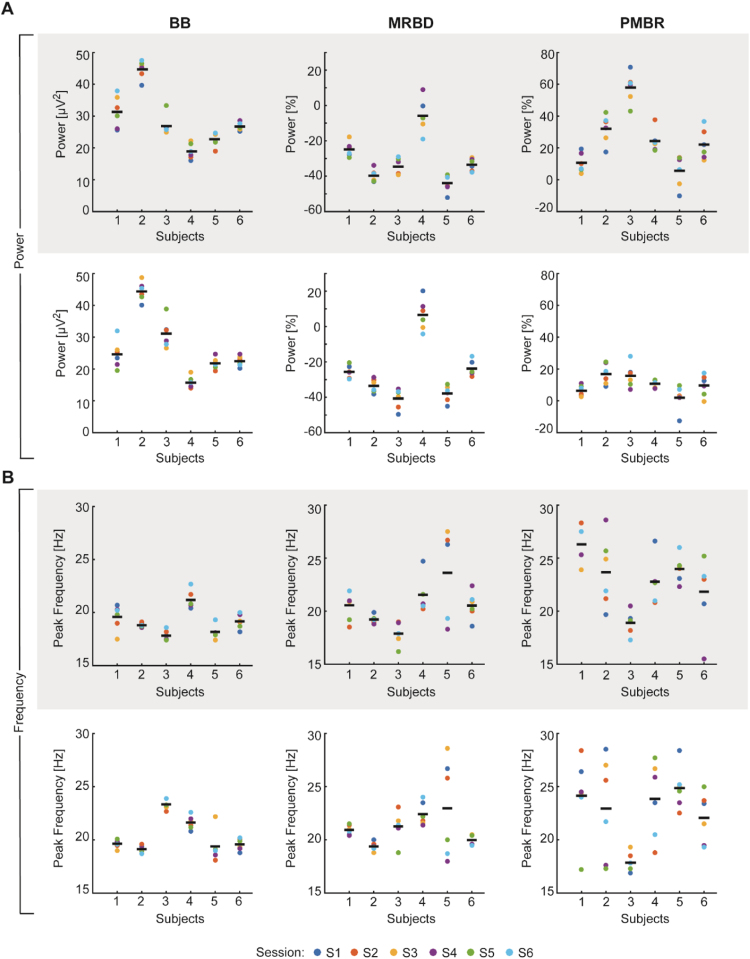
Test-retest reliability of spectral power (A) and peak frequency (B) measures across separate sessions (S1–S6). Individual values were extracted for each EEG session from pre-selected electrodes overlying contralateral (grey shading) and ipsilateral sensorimotor cortex and distinct time windows (BB: 1–0 s; MRBD: 1–2 s; PMBR: 6–7 s). The degree of clustering gives a visual impression of the within-subject and between-subject variation. Black horizontal bars represent grand-mean (across sessions) for each subject.

Fig. 3 shows the pre-movement baseline and movement-related beta parameter estimates derived from contralateral and ipsilateral sensorimotor cortices. The degree of clustering in these plots provides a visual impression of the within- and between-subject variability. Individual baseline beta power ranged approximately 13.87–49.76 µV^2^ in both sensorimotor cortices with an average of 27.6±9.79 µV^2^ (mean ±SD), while within-subject variability was small with a range of 1.19–4.90 µV^2^ (Fig. 4A, left column). The magnitude of MRBD ranged between -52.1 and +20.2% with an average of -30.4 ±14.1% and -25.8±17.5% for contralateral and ipsilateral sensorimotor cortex, respectively (Fig. 4A, middle column). PMBR in contralateral sensorimotor cortex ranged between -10.1 and +70.6% (25.4±19.7%) whereas it only ranged between -12.6 and +28.1% (10.2±7.4%) in ipsilateral sensorimotor cortex (Fig. 4A, right column). By contrast, within-subject variability for MRBD and PMBR power measures was small and fell within a range of ~2–7% per subject.

Individual peak frequencies during the pre-movement baseline period fell within a frequency range of 17.4 to 23.9 Hz (19.8±1.5 Hz) and displayed small within-subject variability of 0.2–1.6 Hz (Fig. 4B, left column). In comparison, peak frequencies of movement-related beta oscillations spanned frequencies from 16.2–29.1 Hz with an average of 20.8±2.2 Hz for MRBD (Fig. 4B, middle column) and 22.7±3.7 Hz for PMBR (Fig. 4B, right column). Notably, within-subject variability was relatively large and ranged from approximately 0.4 –4.8 Hz per subject.

**Fig. 5:**
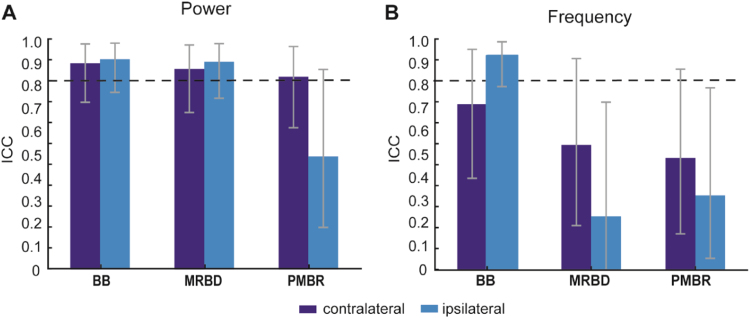
Test-retest reliability of spectral power (A) and peak frequency (B) measures of beta oscillatory activity derived from contralateral and ipsilateral sensorimotor cortices, respectively. Values given are intraclass correlations (ICCs). Grey vertical bars represent lower and upper boundaries of the ICC. ICCs>0.8 indicate almost perfect levels of agreement across sessions.

**Fig. 6:**
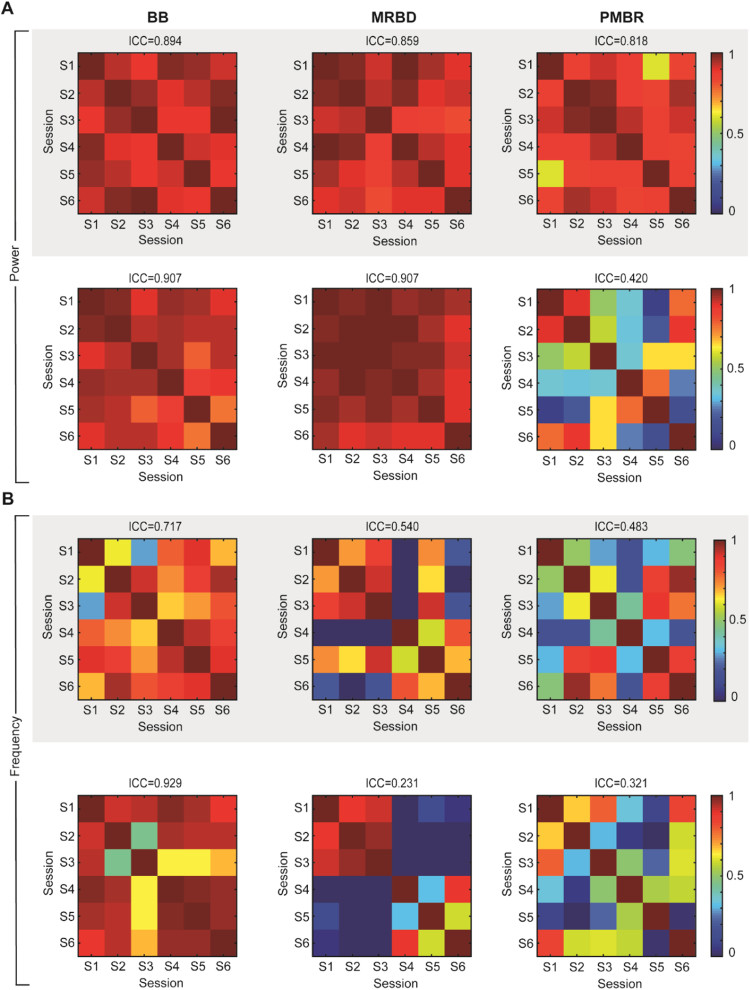
Between-session (S1–S6) correlation coefficients and corresponding intraclass correlation coefficients (ICCs) for spectral power and peak frequency estimates for contralateral (grey shading) and ipsilateral sensorimotor cortices. The colour bar indicates the correlation coefficients (r) presented in the matrices. (For interpretation of the references to color in this figure legend, the reader is referred to the web version of this article).

For a quantitative measure of repeatability of beta oscillations, Intraclass Correlation Coefficients (ICC) were calculated for spectral power of the selected time windows (pre-movement baseline, MRBD and PMBR) and the corresponding peak frequency. Overall, ICC values indicated almost perfect reliability for power measures [mean ICC=0.832, ICC range=0.490–0.912, p<0.001; refer to Fig. 5A], but only moderate reliability for peak frequency estimates [mean ICC=0.537, ICC range=0.231–0.929, p<0.033; refer to Fig. 6B]. ICC values were consistently highest for pre-movement baseline beta power [contralateral sensorimotor cortex: ICC=0.894, p<0.0001; ipsilateral sensorimotor cortex: ICC=0.907, p<0.0001], followed by MRBD [contralateral sensorimotor cortex: ICC=0.859, p<0.0001; ipsilateral sensorimotor cortex: ICC=0.907, p<0.0001] and PMBR power measures [contralateral sensorimotor cortex: ICC=0.818, p<0.0001; ipsilateral sensorimotor cortex: ICC=0.420, p<0.001]. Interestingly, ICC values derived for pre-movement baseline beta and MRBD power estimates yielded slightly higher reliability for ipsilateral than contralateral sensorimotor cortex, while reliability of PMBR power estimates was higher for contralateral sensorimotor cortex. The lower ICC value for PMBR power from ipsilateral sensorimotor cortex was likely due to low between-subject variability, with most values ranging between 0% and 20%, thereby primarily reflecting random fluctuations around the baseline level (Fig. 4A).

Assessment of peak frequency yielded a similar reliability trend, with pre-movement baseline beta peak frequency showing highest ICC values [contralateral sensorimotor cortex: ICC=0.717, *p*<0.0001; ipsilateral sensorimotor cortex: ICC=0.929, *p*<0.001], followed by MRBD [contralateral sensorimotor cortex: ICC=0.540, *p*<0.0001; ipsilateral sensorimotor cortex: ICC=0.231, *p*<0.05] and PMBR peak frequency [contralateral sensorimotor cortex: ICC=0.483, *p*<0.01; ipsilateral sensorimotor cortex: ICC=0.321, *p*<0.01]. Beyond the lower reliability of peak frequency measures compared to spectral power measures of beta activity, movement-related beta peak frequency estimates showed substantially lower reliability, and this appeared to be driven by greater within-subject variability across sessions (Fig. 4B).

In summary, the ICC values indicate that spectral power measures of beta activity were more consistent across EEG sessions than the corresponding peak frequency measures. Additionally, peak frequency during the pre-movement (resting) baseline period was more reliable compared to peak frequency estimates of MRBD and PMBR.

To explore whether test-retest reliability varies as a function of time interval between sessions (i.e. one week apart: session 1–2; two weeks apart: session 1–3; six weeks apart: session 5–6), we calculated Pearson correlation coefficients between each session. Fig. 6 illustrates the correlation coefficients between EEG sessions, separately for spectral power (Fig.6A) and peak frequency (Fig. 6B) measures in the pre-movement baseline (Fig. 6, left column), MRBD (Fig. 6, middle column) and PMBR (Fig. 6, right column) time window. The correlations fluctuated across beta parameter estimates and hemispheres, but no systematic influence of the length of the time interval was observed. Whereas the correlations for pre-movement baseline beta and MRBD power estimates were consistently high across the different test-retest intervals for both contralateral [BBP: *r* range=0.880–0.988, *p* range=0.0002–0.021; MRBD: *r* range=0.880–0.988, *p* range=0.0002–0.021] and ipsilateral sensorimotor cortices [BBP: *r* range=0.750–0.980, *p* range=0.0006–0.060; MRBD: *r* range=0.750–0.980, *p* range=0.0006–0.060], the coefficients for PMBR power showed larger variability, specifically in the ipsilateral [*r* range=0.075–0.900, *p* range=0.014–0.888] compared to the contralateral [*r* range=0.602–0.971, *p* range=0.006–0.207] hemisphere. The notable hemispheric variation in test-retest reliability of PMBR potentially resulted from the absence of an ipsilateral peak in PMBR. While spectral power measures of beta activity demonstrated consistently high between-session correlations, correlation coefficients for peak frequency estimates varied widely. Particularly low coefficients were obtained for movement-related beta activity from contralateral [MRBD: *r* range=-0.427–0.920, *p* range=0.009–0.743; PMBR: *r* range=0.161–0.957, *p* range=0.003–0.760] and ipsilateral [MRBD: *r* range=-0.559–0.954, *p* range=0.003–0.958; PMBR: *r* range=-0.438–0.796, *p* range=0.035–0.916] sensorimotor cortex, while peak frequency of pre-movement baseline beta activity was somewhat more consistent between sessions [contralateral sensorimotor cortex: *r* range=0.285–0.935, *p* range=0.006–0.642; ipsilateral sensorimotor cortex: range=0.439–0.975, p range=0.0009–0.384].

## 4 Discussion

The present study assessed the test-retest reliability of movement-related and pre-movement (resting) beta oscillatory activity in a group of healthy subjects across several weeks. We sought to determine whether EEG-derived spectral power and peak frequency measures of beta oscillations (I) show small within-subject variability and (II) are stable as a function of between-session time interval, two prerequisites for their use as clinically relevant biomarkers. Our results demonstrate that spectral power estimates of resting (BB: average ICC=0.901) and movement-related beta activity (MRBD: average ICC=0.883; PMBR: average ICC=0.619) are remarkably consistent across sessions. In addition, corresponding peak frequency measures yielded lower ICC values compared to the assessment of spectral power. While pre-movement baseline beta peak frequency was highly reliable across sessions, peak frequency measures of movement-related beta activity displayed greater within-subject variability (MRBD: average ICC=0.386; PMBR: average ICC=0.402). The respective between-session correlation coefficients further corroborate these findings. This suggests that measures of spectral power as well as resting peak frequency reflect stable individual activation patterns that could be used to evaluate functional dynamic changes in the brain, such as the impact of disease or treatment administration.

Abundant evidence exists for the reliability of spontaneous resting-state beta activity within the same recording session and between sessions with time intervals of days, weeks and up to years (e.g. Pollock et al. 1991; Burgess and Gruzelier, 1993; Kondacs and Szabo, 1999; McEvoy et al., 2000; Nikulin and Brismar, 2004; Corsi-Cabrera et al., 2007; Napflin et al. 2007; Martin-Buro et al. 2016). However, there is no such literature on movement-related beta oscillations, even though these beta-band dynamics appear to be especially interesting in the study of individual differences related to motor performance. Studies investigating event-related oscillatory activity using cognitive and imagery tasks highlight that their reliability varies as a function of frequency band, brain region and type of task (Friedrich et al., 2013, Krause et al., 2001, Neuper et al., 2005).

Whilst beta oscillations have shown acceptable between-session reliability (Cronbach's alpha >0.7) during motor imagery (Friedrich et al., 2013), little is known regarding reliability during active movements. An indirectly related study from Wilson and colleagues (Wilson et al., 2014) found a linear increase from morning (9:00) to afternoon (16:00) in the amplitude of MRBD and PMBR during a finger tapping task, but small variability over three consecutive days, indicating the reliability of movement-related beta-band signatures. The current study augments the work by Wilson and colleagues by systematically assessing the reliability of spectral power and peak frequency estimates of movement-related beta activity across several weeks.

Compared to previous studies, we used a motor task involving wrist flexion and extension, which are known to elicit stronger PMBR compared to finger and thumb movement (Pfurtscheller et al., 1998b). As a result, consistent with prior findings, we found bilateral suppression of beta oscillatory activity during movement (Gross et al., 2005, Pfurtscheller et al., 1996, Salmelin and Hari, 1994) and clear beta rebound after movement termination, which was significantly larger for contralateral compared to ipsilateral motor cortex (Salmelin and Hari, 1994, Stancak and Pfurtscheller, 1995). Rau et al. (2003) demonstrated that ipsilateral MRBD corresponds to increased cortical excitability of ipsilateral M1, in line with the argument that MRBD indicates activation of the sensorimotor cortex (Pfurtscheller and Berghold, 1989, Pfurtscheller and Lopes, 1999, Rau et al., 2003). However, ipsilateral MRBD has also been proposed to reflect neural processes inhibiting mirror movements through interhemispheric inhibition (Jurkiewicz et al., 2006, Van Wijk et al., 2012). In contrast, PMBR has been associated with inhibition of movement initiation (Gilbertson et al., 2005) in conjunction with decreased corticospinal excitability (Chen et al., 1998). Although the functional role of ipsilateral activity in unimanual motor tasks is not fully understood, the different contra- and ipsilateral modulation patterns for MRBD and PMBR imply that these beta-band dynamics are, at least to a certain degree, independent processes with distinct functional significance.

The high test-retest reliability of movement-related beta power measures suggest that they might be useful in repeated-measures studies, for example, investigating longitudinal changes in clinical and non-clinical populations or assessing the impact of pharmacological interventions. ICC values for MRBD and PMBR estimates were comparably high in both contralateral and ipsilateral sensorimotor cortices, except for PMBR from the ipsilateral hemisphere which was markedly lower. A reliable measure (high ICC) requires small within-subject variance relative to between-subject variance. Closer inspection suggests that the reduced reliability observed for the ipsilateral PMBR was related to the low between-subject variability of this power estimate (see Fig. 4A). In line with previous studies demonstrating a contralateral preponderance of PMBR (Salmelin and Hari, 1994, Stancak and Pfurtscheller, 1995), the ipsilateral PMBR estimates likely reflect random fluctuation around the baseline level, which explains the low between-subject variability and therefore, the lower ICC value.

Individual variability in EEG-derived estimates of beta-band oscillations can be accounted for not only by neural signals of the brain but also by the conductivity of the electrical tissue between the current source and the recording electrode (Buzsáki et al., 2012, Lopes da Silva, 2010). While factors such as pyramidal cell density, cortical microarchitecture, skull thickness and skin conductance affect sensor-derived measures of neuronal oscillations and thus are likely to account for subject-specific differences, they are also expected to be stable over time and therefore also contribute to low intra-individual variability. Accordingly, our findings that test-retest reliability of beta oscillatory activity was independent of between-session time intervals may be attributed to these stable individual differences and a consistent behaviour during the performance of the motor task. However, we note that some of the spectral power measures were less reliable than others (i.e. ipsilateral PMBR), demonstrating that reliability of sensor-derived measures is not solely due to these morphological differences but reflects the variable stability of different neural signals.

Compared to spectral power, peak frequency displayed greater within-subject variability (see Fig. 4B). Although peak frequency during the pre-movement baseline period yielded the highest measures of reliability, test-retest reliability was lower compared to spectral power measures, in particular for contralateral sensorimotor cortex. Peak frequency estimates of MRBD and PMBR displayed fair-to-moderate reliability. Importantly, the reduced test-retest reliability of movement-related beta peak frequency compared to resting peak frequency seems to be related to the active engagement of the motor system. We noted that peak detection for pre-movement baseline beta in some cases was ambiguous when the power spectra showed no clear peak in the beta range even after compensation for the 1/f effect. Furthermore, we found that some subjects displayed double frequency peaks during movement-related beta modulation in line with previous studies suggesting a functional subdivision into low and high frequencies within the beta band (Litvak et al., 2011, Oswal et al., 2016, van Wijk et al., 2016). These factors might be reasons why ICC values were lower.

While measures of beta activity may be affected by a variety of factors, the present study provides evidence that these signatures are highly reliable and consistent over several weeks in a small sample of healthy subjects. The almost perfect intra-individual reliability and high number of sessions provide support to the finding of stable beta power measures. This is important as EEG is an excellent tool for the identification of widely-available and cost-effective biomarkers that might have the potential to bridge the gap between cellular and behavioural accounts of cortical function and plasticity in both healthy and diseased states (Ward, 2015). Establishing the reproducibility of neuronal oscillations is crucial for the identification of EEG-derived biomarkers, with substantial clinical utility for patient stratification and prediction of treatment response.

A potential limitation of this study is the sample of healthy young subjects, which limits the generalisability of the reliability results. In particular, resting and movement-related beta-band estimates have been shown to be modulated by healthy ageing (Gaetz et al. 2010; Rossiter et al., 2014a, Rossiter et al., 2014b; Heinrichs-Graham and Wilson 2016) and pathology (Brown, 2007, Heinrichs-Graham et al., 2013, Heida et al., 2014; Rossiter et al. 2014a) possibly resulting in different reliability patterns. Future studies should thus determine the reliability of movement-related beta-band activity across the lifespan and in the context of movement disorders.

## 5 Conclusion

In conclusion, this study is the first to comprehensively evaluate the reliability of spectral power and peak frequency measures of movement-related beta oscillations across several weeks. The present study highlights that spectral power measures of EEG-derived oscillatory signatures associated with the performance of a motor task are highly reproducible. This finding is important as it suggests that measurements of beta-band power reflect meaningful and reliable individual differences in the motor system that may be utilised as biomarkers in clinical and/or longitudinal research. In addition, our assessments indicate that beta peak frequencies are more variable across sessions which should be taken into account when using extrinsic neurostimulation at beta frequency (Guerra et al., 2016, Joundi et al., 2012, Pogosyan et al., 2009). Overall, the highly reproducible nature of beta oscillations suggests that they may be an appropriate assay for longitudinal studies and/or clinical studies employing sensor-derived EEG-based oscillatory read-outs.

## Conflict of interest statement

There is no conflict of interest.

## Figures and Tables

**Fig. 2:**
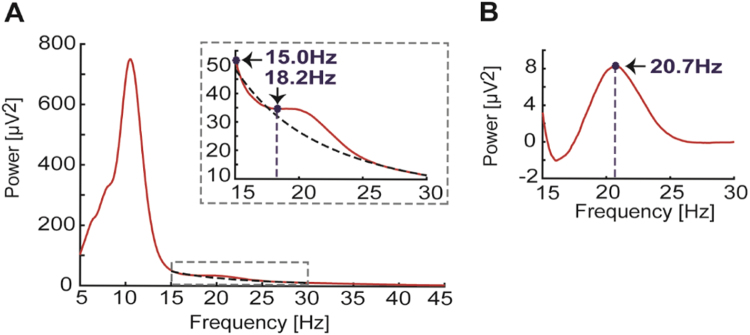
Beta peak frequency detection using least square fit procedure to remove 1/f component from spectrum. A, Power spectrum of one example subject (subject 1) who did not show a clear peak in the beta frequency range (grey dashed rectangle). Black dashed line indicates 1/f component obtained from least square fit of log-log transformed data. Inset shows enlarged view of the spectrum for the beta frequency range. B, Corrected spectrum (after subtraction of 1/f component). Note that in the uncorrected spectrum (A) local maxima were found at 15 Hz or 18.2 Hz, whereas the peak is at 20.7 Hz in B.

**Table 1:** Summary of kinematic measures – reaction time (RT), movement time (MT) and peak velocity (PV) – for each EEG session and condition.

		Session					
	Condition	S1	S2	S3	S4	S5	S6
**RT [ms]**	Flex	529±41	543±48	550±38	536±80	492±53	501±49
	Ext	583±140	583±99	592±134	577±172	518±101	531±124
**MT [ms]**	Flex	905±166	822±162	793±120	767±79	768±92	753±75
	Ext	780±109	664±96	788±158	650±114	660±153	650±139
**PV [deg/s]**	Flex	238±93	238±85	238±57	235±74	257±97	246±76
	Ext	270±107	247±78	226±48	235±87	264±111	268±117

Values given are mean±SD.

**Table 2:** Results of the 6×2×2 ANOVAs for spectral power and peak frequency estimates.

	Time	Condition	Hemisphere	Interactions
**Power**				
BB	*F*_*(5,25)*_=*1.45, p*=*0.240*	*F*_*(1,5)*_=*0.01, p*=*0.958*	*F*_*(1,5)*_=*1.44, p*=*0.284*	all *p>0.2*
MRBD	*F*_*(5,25)*_=*0.77, p*=*0.583*	*F*_*(1,5)*_=*0.46, p*=*0.528*	*F*_*(1,5)*_=*2.68, p*=*0.163*	all *p>0.5*
PMBR	*F*_*(5,25)*_=*1.88, p*=*0.134*	*F*_*(1,5)*_=*1.02, p*=*0.359*	***F***_***(1,5)***_**=*****7.03, p*****=*****0.045***	all *p>0.2*
**Peak frequency**				
BB	*F*_*(5,25)*_=*1.21, p*=*0.341*	*F*_*(1,5)*_=*0.69, p*=*0.454*	*F*_*(1,5)*_=*2.45, p*=*0.192*	all *p>0.4*
MRBD	*F*_*(5,25)*_=*0.35, p*=*0.876*	*F*_*(1,5)*_=*0.99, p*=*0.375*	*F*_*(1,5)*_=*0.63, p*=*0.471*	all *p>0.1*
PMBR	***F***_***(5,25)***_**=*****2.70, p*****=*****0.044***	*F*_*(1,5)*_=*0.00, p*=*0.959*	*F*_*(1,5)*_=*0.09, p*=*0.777*	all *p>0.1*

Significant effects are indicated in bold. BB: pre-movement baseline beta; MRBD: Movement-related beta desynchronization; PMBR: Post-movement beta rebound.
